# Prevalence and Correlates of Families’ Unmet Social Needs in Pediatric Primary Care Settings

**DOI:** 10.3390/healthcare14121671

**Published:** 2026-06-12

**Authors:** Kristen A. Waters, Serena K. Kaul, Sritha R. Donepudi, Sophia D. Danchine, Jennifer M. Hilgeman, Gregory M. Eberhart, John M. Pascoe

**Affiliations:** 1 Boonshoft School of Medicine, Wright State University, Fairborn, OH 45324, USA; 2 Dayton Children’s Hospital, Dayton, OH 45404, USA

**Keywords:** unmet social needs, primary care, social determinants of health, underinsurance, primary caregiver health, childhood chronic health conditions

## Abstract

**Highlights:**

**What are the main findings?**

- Nearly half (47%) of primary caregivers in pediatric primary care settings reported at least one unmet social need, with one-third reporting two or more; the most common needs were food insecurity and inability to pay utility bills.
- In multinomial logistic regression, underinsurance was the strongest independent correlate of reporting ≥2 unmet social needs (AOR = 9.85), followed by household income < $50,000 (AOR = 3.03), positive depression screening (AOR = 2.67), Black race/ethnicity (AOR = 2.02), having a child with special health care needs (AOR = 1.89), and lower caregiver educational attainment (AOR = 1.72).

**What are the implications of the main findings?**

- Underinsurance, low household income, caregiver depression, and race and ethnicity are strongly associated with unmet social needs and are indicators of structural inequities and disparities in healthcare access that must be considered when planning and implementing screenings and community programs.
- The clustering of structural and psychosocial factors among families with two or more unmet social needs, consistent with the Socio-Ecologic Framework, underscores the need for care coordination, resource navigation programs, and community partnerships targeting caregivers to reduce disparities in child health outcomes.

**Abstract:**

**Background/Objectives:** Children of families facing unmet social needs experience higher rates of adverse outcomes compared to those not experiencing unmet social needs. This study aimed to identify factors associated with families’ unmet social needs as reported by parents or guardians at their children’s primary care visits. **Methods**: This cross-sectional study recruited English-speaking primary caregivers of children less than 18 years of age from the Southwestern Ohio Ambulatory Research Network (SOAR-Net) who were surveyed between January 2023 and August 2024. Surveys included the Maternal Social Support Index, Social Capital Scale, RAND Depression Screener, Children with Special Health Care Needs Screener, Medical Expenses of Children Survey, a 10-item social needs screener, and demographics. Data were analyzed with chi-square or Fisher’s exact tests, adjusted logistic regression, and ANOVA. **Results**: Among 1167 respondents (78% response rate), 1114 provided complete data. Primary caregivers were predominantly mothers (79.9%) or fathers (13.6%), White (72.0%) or Black (16.0%), and had an associate’s degree or less (65.1%). The mean (SD) index child’s age was 6.4 (5.3) years, and 52.4% were female. Underinsurance, positive depression screens, and poor child health were positively associated with unmet social needs. Higher scores for social support and social capital were associated with fewer social needs. Multinomial logistic regression revealed significant relationships with reporting two or more unmet social needs with the following variables: childhood underinsurance, household annual income < $50,000, positive depression screens, raising a child with a chronic health condition, and Black race/ethnicity. **Conclusions**: Several significant social factors were independently associated with a greater number of unmet social needs. These findings highlight the complex interplay among social factors in children’s healthcare. Future research should explore the putative longitudinal stability of these relationships.

## 1. Introduction

Unmet social needs (e.g., the absence of basic resources, such as food and housing security, transportation, and health insurance) contribute to the social determinants of health and are well-known to be related to health outcomes [1,2,3,4]. Research interest has grown in recent years regarding the prevalence and sequelae of social determinants of health, illustrating the need for targeted interventions [4,5,6,7,8,9]. The relationship between poverty and children’s health is well documented [10,11,12]. Furthermore, adverse childhood experiences are known to “cast a long shadow” [10] and affect adults’ health [5,6,10,11,12].

Prior studies document associations between social determinants of health and adverse outcomes in adults, though pediatric research focuses more on individual domains such as food insecurity and housing instability and not multiple unmet social needs within child populations [7,8,9,13,14]. There have also been relatively fewer studies on psychosocial and demographic correlates associated with unmet social needs specifically within community-based primary care settings [15,16]. Therefore, gaps remain in understanding how various social needs conglomerate to impact families’ health outcomes [1,4].

Profound barriers to adequate resources to support pediatric health include a myriad of unmet social needs that were analyzed in this study. Underinsurance is associated with delayed preventive care, lower quality of care, and an increased rate of unmet health needs [17]. These challenges are often magnified for children with special health care needs (CSHCN), whose families face disproportionately high medical barriers and out-of-pocket costs when living in low- to middle-income households [18]. Other psychosocial stressors analyzed in this study, such as primary caregiver depression, interact with these structural inequities to further compromise child health and limit parents’ capacity to foster healthy environments for their children [19]. While associations among social determinants of health and health outcomes are well-established in adults, research that examines social factors, including unmet social needs among children’s caregivers, remains limited.

Screening and addressing unmet social needs in children’s health care settings improves health outcomes for children [15,16,20,21]. A randomized clinical trial found that families who receive in-person assistance to access social services had a significant decrease in the number of social needs reported and significantly greater improvement in their child’s health compared to families who solely received written information on community resources [22]. Understanding the psychosocial correlates of unmet social needs may inform and improve screening practices and interventions in child health care settings [21,23]. Primary care child health clinicians play an integral role in the physical and mental health of the children they serve, including the social needs of families that affect children’s overall wellbeing [1].

Practice-based research networks (PBRNs) have played an increasingly prominent role in primary care research in the United States [23,24,25]. These networks consist of groups of practices that participate in the investigation of clinical practice phenomena in their communities. The Southwestern Ohio Ambulatory Research Network (SOAR-Net) includes child health clinicians from economically and geographically diverse areas in Dayton and Springfield, Ohio [25,26,27].

This study aims to estimate the prevalence of families’ unmet social needs reported by families in southwest Ohio through a PBRN and examine correlates of the reported unmet social needs in child health primary care settings. Evaluating multiple categories of unmet social needs together within a community-based population aims to extend prior research beyond single correlate prevalence estimates and provide insight into the broader social vulnerabilities that families face. These findings will enhance our understanding of the social needs of families in southwestern Ohio and their relation to child health in the community [10].

## 2. Materials and Methods

### 2.1. Study Population

This study used recruitment methods similar to other previously published SOAR-net studies and other primary care research network studies [24,26,27]. Primary caregivers of index children were recruited in the waiting rooms of participating practices. Primary caregivers were eligible if they were literate in English and the child they accompanied was less than 18 years of age. Respondents included mother, father, grandparent, and other caregivers. If multiple children were being seen at one appointment, primary caregivers were asked to complete the survey using the youngest child as the subject. Surveys were not included in the analysis if any of the following applied: the respondent was not the primary caregiver, the relation to the child was missing, the child’s age was not provided or was outside the study methods range (less than 18 years), or more than 2 of the 10 social needs resource questions were missing or unanswered. After conducting a preliminary pilot study using 30 questionnaires, study data collection was initiated in January 2023 and continued through August 2024.

### 2.2. Study Sites

The Southwestern Ohio Ambulatory Research Network (SOAR-Net) is a primary care practice-based research network created in 2002 [10,23,26,27]. It includes 9 practices located in economically and geographically diverse areas of the Miami Valley of Ohio [27]. This study was conducted at 9 SOAR-Net practices, and all participating practices agreed to a minimum of 1 week of recruitment time [27].

### 2.3. Study Questionnaire

The survey consisted of 87 questions and was developed from several validated questionnaires. It was self-administered, anonymous, and voluntary. It included demographic information, the Medical Expenses for Children Survey (MEoCS) [26,27,28,29], the Maternal Social Support Index (MSSI) [30], RAND Depression Screener [31], the Social Capital Scale (SCS) [32], Children with Special Health Care Needs (CSHCN) [33], and primary caregiver and index child demographic information. To protect participant confidentiality, social needs identified through survey responses were not individually addressed; instead, all families received information about United Way 211, a community resource referral service, to explore relevant local supports at their own discretion [34,35].

### 2.4. Unmet Social Needs

The survey assessed primary caregivers’ and index children’s demographics, their social needs, underinsurance, health status of the index child, and primary caregivers’ social support and social capital. Social needs questions were adapted from social needs screening questionnaires of a local children’s hospital and a federally qualified health center, which were adapted from the Center for Medicare and Medicaid Services [36]. The screener assessed 10 items across the following domains: food insecurity (2 items), transportation (2 items), housing/shelter instability, safety, utilities, household items, child-related resources, and other needs (open-ended). Each closed-ended item was coded as a binary variable, with responses of “Sometimes” or “Often” coded as an unmet need (1) and “Never” coded as no unmet need (0). Each item was counted separately toward the total score, resulting in a maximum possible total score of 10. The total number of unmet social needs was calculated as the sum of endorsed items. Participants were then categorized as having 0, 1, or 2 or more unmet social needs for analysis.

### 2.5. Medical Expenses for Children Survey (MEoCS)

The Medical Expenses for Children Survey (MEoCS) has been utilized in similar SOAR-Net research since 2009 [26,27,28,29]. It is an adaptation of the High Plains Research Network Community Council validated Voorhees’ Medical Expenses Survey, which utilized seven questions to determine whether adult respondents were underinsured [27,28]. The colonoscopy screening question was deemed irrelevant for children, and it was omitted. However, the remaining six questions were included in this study, and the response options remained unchanged (yes, no, and don’t know).

### 2.6. Maternal Social Support Index (MSSI)

The MSSI is a 21-item questionnaire that assesses aspects of personal social support [30]. The total score ranges from 0–39, and higher scores are associated with greater social support. The survey was used in its entirety for this analysis.

### 2.7. RAND Depression Screener

The RAND Depression Screener was used to assess depressive symptoms. This instrument was developed by the RAND Corporation for the Partners in Care study and includes items adapted from the Composite International Diagnostic Interview (CIDI) along with additional self-report symptom questions [31]. A participant was considered to have a positive depression screen if 2 of 3 questions were positive. The screener demonstrated moderate predictive validity, with approximately 55% of those screening positive meeting criteria for a depressive disorder on the full CIDI interview. The questionnaire and scoring instructions are publicly available on the RAND Corporation website [31].

### 2.8. Social Capital Scale (SCS)

The SCS is a 20-item questionnaire that assesses an individual’s perception of their community and its social capital [32]. The questionnaire assesses community involvement, informing, asking, and belonging in the community. The score ranges from 20–100, with higher scores associated with greater community social capital. The survey was used in its entirety for this study.

### 2.9. Children with Special Health Care Needs (CSHCN)

The CSHCN Screener is a set of five questions used to identify children with chronic or special health care needs [33]. The survey was used in its entirety in this study. There are three domains: (1) Dependency on prescription medications, (2) Service use above that considered usual or routine, and (3) Functional limitations [37]. Responses classify children as (1) no special health care needs, (2) limitations in dependency needs only, (3) service use needs only, (4) functional only, (5) dependency and service use, (6) dependency and functional, (7) service use and function, and (8) dependency, service use, and functional. This classification system was employed for this study and merged some categories for analysis.

### 2.10. Demographics

Demographic questions for both primary caregivers and index children were included in the survey. Survey respondents were asked if they were the primary caregiver of the child at the appointment—the index child. Respondents reported their marital status and relation to the index child and answered questions about their and the index child’s race, sex, education, insurance status, household income, and their perception of the child’s overall health.

To determine the index child’s healthcare underinsurance status, primary caregivers were asked if, in the last 12 months, due to inability to pay, (1) they had delayed care for their child, (2) were unable to make or keep an appointment for their child, or they were unable to (3) take their child to see a specialist, (4) obtain a test, (5) fill a prescription, or (6) get other medical care for their child that a clinician recommended. A child was classified as underinsured if their primary caregiver responded “yes” to 1 or more of these questions.

### 2.11. Statistical Analysis

Surveys, 53 in number, were excluded as described in Section 2.1. Analyses were performed on the remaining 1114 surveys. Methods for statistical analysis followed the similar protocols of previously published SOAR-net studies [10,26,27].

Descriptive statistics included mean and standard deviation (SD) for continuous variables and frequency (percent of non-missing responses) for categorical variables. Categorical variables were analyzed using chi-square or Fisher’s exact tests to determine unadjusted associations among independent variables and unmet social needs status. Multinomial logistic regression analysis was performed to calculate the adjusted odds ratios and 95% confidence intervals for factors associated with the number of unmet social needs. All analyses were conducted with IBM SPSS Statistics for Windows, v29.0 (IBM Corporation, Armonk, NY, USA). *p* values < 0.05 were considered statistically significant. Variables were selected for inclusion in the multinomial logistic regression based on statistical significance (*p* < 0.05) in bivariate analyses. The following variables were excluded due to lack of bivariate statistical significance: site ID, child’s sex, child’s race, child’s previous insurance, household substance use, and firearm storage in the home. Where multiple operationalizations of a variable were available, the most parsimonious form was retained. Specifically, a binary operationalization of CSHCN status was used in place of the 3-level and 8-level versions, a binary operationalization of child’s insurance status was used in place of the 3-level version, and the Social Capital Scale total score was used in place of individual subscale scores. Variables that were redundant with composite measures already included in the model were also excluded, including the individual component items of the depression screener, the individual component items of the underinsurance measure, and the item assessing change in ease of accessing care over time. Complete-case analysis was applied across all variables retained in the final model.

## 3. Results

Primary caregivers numbering 54% reported 0 unmet social needs, while 13% had 1 unmet social need, and 34% had ≥2 unmet social needs. The mean number (SD) of unmet social needs for all participants was 1.5 (2.1). Primary caregivers with ≥2 unmet social needs reported a mean of 3.9 (1.7) unmet needs, a median of 4.0 (2.0), and a range of 2 to 10 unmet needs. Primary caregivers were predominantly mothers (79.9%) or fathers (13.6%), White (72.0%) or Black (16.0%), and had an associate’s degree or less (65.1%). The mean index child’s age was 6.4 (5.3) years, and 52.4% were female.

Table 1 presents the demographic characteristics of study participants stratified by unmet social needs groups: 0, 1, and ≥2 unmet social needs. Significant demographic differences were observed across unmet social needs categories (*p* < 0.001 for most variables). Among primary caregiver demographics, Black caregivers, those with lower educational attainment (associate’s degree or less), those reporting household annual incomes below $50,000, and those who were single, separated, divorced, or widowed were more likely to have ≥1 unmet social needs. Mothers comprised the majority of caregivers across all groups (Table 1). Index children who were identified as Black, had public insurance, or had fair or poor overall health ratings were more likely to have one or more unmet social needs compared to index children whose primary caregivers reported no unmet social needs (Table 1). No significant differences were observed by child gender across unmet social needs groups (*p* = 0.715).

Overall rates for the most commonly reported unmet social needs were as follows: food running out before the participant had resources to get more (30.8%), food not lasting or not having resources to get more (28.0%), and inability to pay for utility bills (26.1%). Significant associations were found between social variables and the number of unmet social needs reported. Among primary caregivers, those raising children with service use limitations or functional limitations per the CSHCN screener had a higher proportion of ≥2 unmet social needs compared to 0 or 1 unmet social needs (46% and 57%, respectively; *p* < 0.001 for both; Table 2). Similarly, a higher proportion of caregivers with a positive underinsurance screen reported 2 or more unmet social needs (70%) compared to 1 (13%) unmet social need (*p* < 0.001; Table 2). Caregivers with positive depression symptoms reported 2 or more unmet social needs at a higher proportion (56%) compared to 1 (10%) or 0 (34%) unmet social needs (*p* < 0.001; Table 2).

The number of social needs reported was inversely related to markers of social support (MSSI score) and social capital (SCS score) (Table 2). Both the mean MSSI Total and mean SCS Total scores differed significantly across groups (*p* < 0.001), with MSSI scores decreasing as unmet needs increased (from 25 to 19) while SCS scores also declined (from 69 to 62). There was no significant relationship between the number of social needs reported and substance use in this study population (*p* = 0.341).

A multinomial logistic regression model was constructed to determine the odds of having two or more unmet social needs versus no unmet social needs (Table 3). The regression analysis included 834 participants (74.9% of the total sample), with 473 (56.7%) having no unmet social needs and 266 (31.9%) having two or more unmet social needs. For the one versus no unmet social needs comparison, no variables reached statistical significance after adjustment with the exception of fair/poor overall child health status (AOR = 3.98, 95% CI: 1.19, 13.27, *p* = 0.025), indicating that caregivers reporting fair or poor child health had approximately four times greater odds of reporting one unmet social need compared to no unmet social needs (Supplementary Table S3). All other variables included in the model did not reach statistical significance for this comparison (all *p* > 0.05).

However, several factors emerged as significant associations with having two or more unmet social needs, when adjusted for the following variables: underinsurance, household income, depression screening, PCG race, CSHCN, PCG education, child overall health status, social capital, primary caregiver marital status, maternal social support, primary caregiver age, and child insurance type (Table 3). Being underinsured was the strongest association (AOR = 9.85, 95% CI: 4.05–23.93), followed by household income below $50,000 (AOR = 3.03, 95% CI: 1.72–5.32), positive depression screening (AOR = 2.67, 95% CI: 1.80–3.97), and Black race (AOR = 2.02, 95% CI: 1.15–3.55). Additional significant variables included having a child with special health care needs (AOR = 1.89, 95% CI: 1.22–2.92) and primary caregiver education of associate’s degree or less (AOR = 1.72, 95% CI: 1.02–2.90). Other factors, including the child’s overall health, social capital, primary caregiver marital status, maternal social support, primary caregiver age, and child’s insurance type had adjusted 95% confidence intervals that included 1.0.

## 4. Discussion

This study examined the prevalence and correlates of unmet social needs among caregivers of children in primary care settings. Nearly half of caregivers reported at least one unmet social need, and one-third reported two or more unmet needs, highlighting the substantial burden of social vulnerability in this population. These findings are consistent with prior studies that reported a high prevalence of unmet social needs among children’s families in primary care venues and reinforce the importance of screening within clinical settings [15,16,21]. This analysis identified several sociodemographic characteristics through bivariate analysis that were significantly associated with reporting one or more unmet social needs: Black race/ethnicity, lower caregiver educational attainment (associate’s degree or less), annual household income below $50,000, and unmarried status of caregiver (including single, separated, divorced, or widowed individuals). In addition, caregivers of children with chronic health conditions, caregivers raising children experiencing underinsurance, and caregivers screening positive for symptoms of depression were significantly more likely to report multiple unmet social needs.

The findings of this study highlight the multidimensional and intersecting nature of social determinants of health among vulnerable populations, illustrating that social needs rarely occur in isolation. Rather, this study showed that the odds of experiencing two or more unmet needs are significantly related to factors rooted in structural and systemic disadvantage. The connection between socioeconomic vulnerability and child health is well established [4,38,39,40,41,42,43]. Children’s adherence to medical regimens and overall health are dependent on the stability, resources, and knowledge of their caregivers, thus social vulnerability within the family environment directly translates to clinical risk for the child [39,41,44,45]. Caregivers facing financial strain, inadequate health insurance coverage, or mental health challenges may experience difficulty adhering to treatment plans, attending follow-up visits, or accessing recommended care for their child [41]. These challenges may be amplified for families managing chronic childhood health conditions [33]. Such structural inequities align with the Socio-Ecologic Framework, demonstrating that individual health outcomes (Individual/Interpersonal Level) are encompassed within and fundamentally guided by community (Organizational/Community Level) and policy-related (Public Policy Level) disadvantages [46,47]. Moreover, given the complexity of factors related to health outcomes, it is not surprising that this analysis shows that families facing multiple concurrent social challenges often have the highest rates of unmet social needs.

The strong associations observed in this study among caregiver positive depression symptoms, child underinsurance, and multiple unmet needs of the family further highlight the importance of screening for psychosocial and social factors together rather than in isolation. Specific factors studied that emerged as correlates of unmet social needs help address a critical gap in the existing child health literature, particularly within primary care settings. The high prevalence of food insecurity and utility needs observed in this study mirrors national data and emphasizes the importance of routine screening for these domains in child health practice [4,13,14]. Screening programs that incorporate resource navigation, care coordination, and community partnerships help address these needs and improve child health outcomes [15,20,22,38].

This study has several notable strengths. First, it examined a socioeconomically diverse population of caregivers recruited from primary care child health settings, enhancing external validity and clinical applicability. Second, the utilization of validated and common social needs screening instruments strengthened the methodological rigor and mirrors real-world clinical implementation. This provides support for the practical utility of such tools in primary care contexts. The large sample size and inclusion of multiple psychosocial variables facilitated a comprehensive assessment of correlates of unmet social needs.

However, several limitations warrant consideration. The cross-sectional design precludes causal inference regarding the relationships among sociodemographic factors and unmet social needs. In addition, self-report bias represents a potential threat to validity, though this was partially mitigated with psychometrically validated screening measures. Recruitment was limited to English-speaking caregivers, which may restrict the generalizability of findings to non-English speaking populations, a demographic that prior research demonstrated by experiencing disproportionately elevated rates of unmet social needs [48]. Additionally, the study was conducted in a single geographic region, which may induce a sampling bias and limit generalizability to other settings.

Several methodological limitations warrant consideration. The social needs screener included two items each for food insecurity and transportation; each counted separately toward the total score, potentially giving these domains greater weight relative to single-item domains and influencing participant categorization near scoring thresholds. Participants missing 1 or 2 social needs responses were retained in the analysis with missing items coded as not endorsed, which may have slightly underestimated the true burden of unmet social needs. Variable selection for the multinomial logistic regression was based on bivariate statistical significance (*p* < 0.05), and where multiple operationalizations were available, the most parsimonious form was selected (e.g., composite scores, binary CSHCN status). The resulting reduction in sample size from 1114 to 834 participants, combined with the use of complete-case analysis, may have introduced bias if the missing data were not missing completely at random, and regression findings should be interpreted accordingly.

Future research should employ longitudinal and mixed method designs to elucidate causal pathways between identified correlates and unmet social needs. Particularly valuable would be qualitative or quantitative investigations that examine the lived experiences of caregivers of children with chronic health conditions and other marginalized populations, which may inform more targeted and culturally appropriate intervention strategies. Implementation studies that evaluate integrated screening models, resource navigation programs, and primary care-based interventions may help identify effective strategies to address unmet social needs in families raising children [13].

## 5. Conclusions

In this primary care cohort, nearly half of caregivers reported one or more unmet social needs. Families with multiple unmet needs were more likely to experience underinsurance, lower household income, lower levels of social support, and higher levels of positive depressive symptoms. Social factors, such as respondents who identified as Black and caregivers for children with special health care needs, further increased the likelihood of reporting multiple unmet needs, highlighting the interrelated social and psychosocial factors that contribute to child health disparities. These findings also underscore the importance of routine social needs screening in child health primary care settings. Integrating screening for caregiver mental health, insurance barriers, and social needs may improve identification of families at highest risk. Strengthening partnerships with community resources and implementing care coordination or resource navigation programs may help address unmet needs and reduce disparities in child health outcomes. Future studies should explore the longitudinal, potentially dynamic, patterns of unmet needs and their correlates and evaluate targeted primary care interventions designed to address unmet social needs and improve child health outcomes.

## Figures and Tables

**Table 1:** Demographic factors associated with unmet social needs reporting.

Demographic Variable	Level	Total	Number of Unmet Social Needs			*p*	Significance by Group
			0	1	≥2		
Primary Care Giver Demographics							
Race (*n* = 1060)	White	763	59%	12%	30%	<0.001	1 vs. 0; ≥2 vs. 0
	Black	170	33%	15%	52%		
	Other/Multiracial	127	56%	9%	35%		
Education (*n* = 1071)	≥College grad	374	74%	13%	13%	<0.001	≥2 vs. 0; ≥2 vs. 1
	≤AA/Some college	697	43%	12%	45%		
Household Income (*n* = 1034)	≥$50,000	524	73%	12%	16%	<0.001	All groups
	<$50,000	510	35%	13%	52%		
Marital Status (*n* = 1070)	Married or Unmarried Couple	699	64%	11%	25%	<0.001	All groups
	Single, Separated, Divorced, or Widowed	371	35%	14%	51%		
Relation to Child (*n* = 1072)	Mother	857	52%	13%	36%	0.001	≥2 vs. 0
	Father	146	69%	12%	19%		
	Grandparent	35	40%	11%	49%		
	Other	34	53%	9%	38%		
Index Child Demographics							
Race (*n* = 1047)	White	654	60%	13%	27%	<0.001	≥2 vs. 0
	Black	175	37%	14%	49%		
	Other/Multiracial	218	52%	9%	39%		
Insurance Type (*n* = 1112)	Public	614	39%	13%	49%	<0.001	All groups
	Private	483	73%	12%	15%		
	None	15	40%	20%	40%		
Overall Health (*n* = 1097)	Excellent, Very Good, or Good	1061	55%	12%	34%	<0.001	1 vs. 0; ≥2 vs. 0
	Fair or Poor	36	25%	28%	47%		

Survey totals are listed under each demographic variable and may differ due to missing or incomplete information in some surveys. The Total column represents the count of respondents for each level. Rates represent percentage of total surveys for each level that reported 0, 1, or ≥2 unmet social needs. Percents may not be equal to 100 due to rounding. *p*-values are from chi-square or Fisher’s exact tests and reflect overall group differences. *p*-values < 0.05 are considered significant. Only significant associations through bivariate analysis are included. Full bivariate analysis is available in Supplementary Table S1. For multiple comparisons among groups, *p*-values were adjusted with Bonferroni corrections for 3 multiple comparisons. The “Significance by Group” column indicates which subgroup comparisons were statistically significant in post hoc testing (0 unmet needs vs. 1 unmet need; 0 vs. ≥2 unmet needs; etc.).

**Table 2:** Factors related to unmet social needs.

Variables	All Surveys	Number of Unmet Social Needs			*p* Value	Significance by Group
		0	1	≥2		
CSHCN						
Dependency	217	45%	12%	43%	0.002	≥2 vs. 0
Service Use	281	43%	12%	46%	<0.001	≥2 vs. 0; ≥2 vs. 1
Functional Limitations	118	34%	9%	57%	<0.001	≥2 vs. 0; ≥2 vs. 1
Positive Underinsurance Screen	70	17%	13%	70%	<0.001	≥2 vs. 1; 1 vs. 0
Positive Depression Screen	353	34%	10%	56%	<0.001	≥2 vs. 0; ≥2 vs. 1
Mean MSSI Total	23 (7)	25 (6)	23 (6)	19 (7)	<0.001	All groups
Mean SCS Total	66 (10)	69 (9)	67 (10)	62 (9)	<0.001	All groups

Counts are reported for all surveys. Rates of 0, 1, or ≥2 unmet social needs are reported for each categorical variable. Mean values for continuous variables are reported with standard deviations in parentheses. *p* values are from chi-square tests (c) or Fisher’s exact tests (f). For multiple comparisons between paired groups, *p* values were adjusted with Bonferroni corrections for 3 multiple comparisons. Percents may not total exactly 100 due to rounding. “Significance by group” indicates which subgroups were statistically significant. Only significant associations through bivariate analysis are included. Full bivariate analysis is available in Supplementary Table S2. Abbreviations: Maternal Social Support Index (MSSI), Social Capital Scale (SCS), Children with Special Health Care Needs (CSHCN).

**Table 3:** Multinomial logistic regression illustrating the odds of reporting ≥ 2 unmet social needs versus no unmet social needs.

Variable	*n*	≥2 Unmet Social Needs Adjusted Odds Ratio (95% Confidence Interval)
Underinsured		
Yes	45	**9.85 (4.05, 23.93)**
No	789	Reference Group
Household Income		
<$50,000	390	**3.03 (1.72, 5.32)**
≥$50,000	444	Reference Group
Depression Screen		
Positive	273	**2.67 (1.80, 3.97)**
Negative	561	Reference Group
PCG Race		
Black	111	**2.02 (1.15, 3.55)**
Other/Multiracial	100	0.69 (0.38, 1.24)
White	623	Reference Group
CSHCN		
CSHCN1	267	**1.89 (1.22, 2.92)**
Not CSHCN	567	Reference Group
PCG Education		
≤Associate/Some college	516	**1.72 (1.02, 2.90)**
≥College grad	318	Reference Group

The sample size for the regression is 834 (74.9% of total sample size of 1114): 473 of 834 (56.7%) have 0 unmet social needs and 266 (31.9%) have ≥2 unmet social needs. For the one versus no unmet social needs comparison, no variables reached statistical significance after adjustment, with the exception of fair/poor overall child health status, which was associated with approximately four times greater odds of reporting one unmet social need compared to no unmet social needs (AOR = 3.98, 95% CI: 1.19, 13.27, *p* = 0.025). Full results for the one versus no unmet social needs are available in Supplementary Table S3. Significance is denoted by bolding. Adjusted odds ratios (AORs) are adjusted for all variables presented in the table, as well as the following covariates excluded from the table due to non-significant associations (95% CI inclusive of 1.0): child overall health status, social capital, primary caregiver marital status, maternal social support, primary caregiver age, and child insurance type. Abbreviations: Primary Caregiver (PCG), Children with Special Health Care Needs (CSHCN).

## Data Availability

Because participant confidentiality was assured at the time of data collection, individual-level data cannot be shared publicly. Aggregated data supporting the reported results may be made available upon reasonable request to the corresponding author.

## Supplementary Materials

The following supporting information can be downloaded at https://www.mdpi.com/article/10.3390/healthcare14121671/s1. Table S1—Demographic and Unmet Social Need Variable Analysis; Table S2—Correlate Variable Analysis; Table S3—Multiple Logistic Regression Analysis.

## Abbreviations

The following abbreviations are used in this manuscript:

SOAR-Net	Southwestern Ohio Ambulatory Research Network
PBRN	Practice-Based Research Network
MEoCS	Medical Expenses for Children Survey
MSSI	Maternal Social Support Index
RAND	Research ANd Development (Corporation)
SCS	Social Capital Scale
CSHCN	Children with Special Health Care Needs
CIDI	Composite International Diagnostic Interview
SD	Standard Deviation
ANOVA	Analysis of Variance
AOR	Adjusted Odds Ratio
CI	Confidence Interval
SPSS	Statistical Package for the Social Sciences
PCG	Primary Caregiver
CMS	Center for Medicare and Medicaid Services
