# Supplementary material for: Prevalence and Correlates of Families’ Unmet Social Needs in Pediatric Primary Care Settings
Source: Healthcare (Basel). 2026 Jun 12;14(12):1671. doi: 10.3390/healthcare14121671 (PMC13299676; doi:10.3390/healthcare14121671)
Supplement: Supplementary file 1 [file healthcare-14-01671-s001.zip › Waters_S1_Demographics.pdf]

Supplemental Table S1: Summary of demographic and unmet social needs screening information. The data summary includes 1114 of 1167 surveys (95.5%) collected from 01/20/2023 through 08/15/2024. Survey respondents are the primary caregivers (PCGs) (all relationships included) of children aged <18 years.

a) Reasons for survey exclusion.

Fifty-three surveys were excluded for the following reasons:	n (%) of 53	n (%) of 1167
PCG not primary caregiver or primary caregiver missing	35 (66.0)	35 (3.0)
Relation to child missing	5 (9.4)	5 (0.4)
Child's age missing	2 (3.8)	2 (0.2)
Child's age ≥18 years	5 (9.4)	5 (0.4)
Missing >2 of 10 resources questions	6 (11.3)	6 (0.5)
Total	53 (100)	53 (4.0)

Instruments used in the study:
CSHCN: Children with Special Health Care Needs screener
Depression screen
MSSI: Maternal Social Support Index (scored if missing <=1)
SCS: Social Capital Scale
Social Needs questionnaire
Underinsurance screen

b) Social Needs Questionnaire individual social needs/resources: Of 1114 surveys, 597 (53.6%) had no unmet social needs; 140 (12.6%) had one unmet social need; 377 (33.8%) had ≥two unmet social needs.

In the past 12 months:	Level	All surveys n (%)	PCGs with ≥one unmet social needs n (%)	PCGs with one unmet social need n (%)	PCGs with ≥two unmet social needs n (%)
The food you bought just didn't last and you didn't have money to get more.	Never	801 (72.0)	204 (39.5)	121 (86.4)	83 (22.1)
	Sometimes	266 (23.9)	266 (51.6)	17 (12.1)	249 (66.2)
	Often	46 (4.1)	46 (8.9)	2 (1.4)	44 (11.7)
	Total	1113 (100)	516 (100)	140 (100)	376 (100)
You worried that the food you bought would run out before you got the money or food stamps to buy more.	Never	770 (69.2)	173 (33.6)	116 (82.9)	57 (15.2)
	Sometimes	278 (25.0)	278 (54.0)	23 (16.4)	255 (68.0)
	Often	64 (5.8)	64 (12.4)	1 (0.7)	63 (16.8)
	Total	1112 (100)	515 (100)	140 (100)	375 (100)
Lack of transportation has kept you from meetings, work, running errands, or getting things needed for daily living.	Never	987 (89.0)	392 (76.3)	133 (97.1)	259 (68.7)
	Sometimes	91 (8.2)	91 (17.7)	4 (2.9)	87 (23.1)
	Often	31 (2.8)	31 (6.0)	0 (0.0)	31 (8.2)
	Total	1109 (100)	514 (100)	137 (100)	377 (100)

Lack of transportation has kept you from medical appointments or from getting medications.	Never	1022 (92.0)	427 (82.8)	139 (99.3)	288 (76.6)
	Sometimes	73 (6.6)	73 (14.1)	1 (0.7)	72 (19.1)
	Often	16 (1.4)	16 (3.1)	0 (0.0)	16 (4.3)
	Total	1111 (100)	516 (100)	140 (100)	376 (100)
You were worried about where you and your family would spend the night.	Never	1093 (98.3)	496 (96.3)	140 (100)	356 (94.9)
	Sometimes	13 (1.2)	13 (2.5)	0 (0.0)	13 (3.5)
	Often	6 (0.5)	6 (1.2)	0 (0.0)	6 (1.6)
	Total	1112 (100)	515 (100)	140 (100)	375 (100)
You felt physically or emotionally UNSAFE in your home.	Never	1084 (97.5)	489 (94.6)	137 (97.9)	352 (93.4)
	Sometimes	25 (2.2)	25 (4.8)	3 (2.1)	22 (5.8)
	Often	3 (0.3)	3 (0.6)	0 (0.0)	3 (0.8)
	Total	1112 (100)	517 (100)	140 (100)	377 (100)
You were worried about or unable to pay your utility bills (gas, electric, phone, etc.).	Never	823 (73.9)	227 (43.9)	108 (77.1)	119 (31.6)
	Sometimes	251 (22.6)	251 (48.5)	31 (22.1)	220 (58.4)
	Often	39 (3.5)	39 (7.5)	1 (0.7)	38 (10.1)
	Total	1113 (100)	517 (100)	140 (100)	377 (100)
You were worried about or unable to find necessary household items (appliances, furniture, etc.).	Never	962 (86.6)	365 (71.0)	138 (100)	227 (60.4)
	Sometimes	132 (11.9)	132 (25.7)	0 (0.0)	132 (35.1)
	Often	17 (1.5)	17 (3.3)	0 (0.0)	17 (4.5)
	Total	1111 (100)	514 (100)	138 (100)	376 (100)
You were unable to get child related resources (educational support for your children, childcare, diapers, clothing, car seats, cribs, etc.).	Never	981 (88.3)	384 (74.7)	136 (97.1)	248 (66.3)
	Sometimes	108 (9.7)	108 (21.0)	2 (1.4)	106 (28.3)
	Often	22 (2.0)	22 (4.3)	2 (1.4)	20 (5.3)
	Total	1111 (100)	514 (100)	140 (100)	374 (100)
Are there any other things you have needed help with?	Never	960 (87.5)	367 (72.8)	84 (61.3)	283 (77.1)
	Sometimes	137 (12.5)	137 (27.2)	53 (38.7)	84 (22.9)
	Often	- (-)	- (-)	- (-)	- (-)
	Total	1097 (100)	504 (100)	137 (100)	367 (100)

c) Number of Unmet Social Needs.

Variable	Statistic	All surveys	PCGs with no unmet social needs	PCGs with one unmet social need	PCGs with ≥two unmet social needs
Number of unmet social needs (resources) (of 10)	Mean (SD)	1.5 (2.1)	3.1 (2.0)	1.0 (0.0)	3.9 (1.7)
	Median (IQR)	0.0 (3.0)	3.0 (3.0)	1.0 (-)	4.0 (2.0)
	Range	0-10	1-10	1.0	2-10
	n	1114	517	140	377

IQR = interquartile range; SD = standard deviation

In the past 12 months:	Level	All surveys n (%)	PCGs with ≥one unmet social needs n (%)	PCGs with one unmet social need n (%)	PCGs with ≥two unmet social needs n (%)
Number of unmet social needs (resources) (of 10)	0	597 (53.6)	- (-)	- (-)	- (-)
	1	140 (12.6)	140 (27.1)	140 (100)	- (-)
	2	89 (8.0)	89 (17.2)	- (-)	89 (23.6)
	3	94 (8.4)	94 (18.2)	- (-)	94 (24.9)
	4	78 (7.0)	78 (15.1)	- (-)	78 (20.7)
	5	55 (4.9)	55 (10.6)	- (-)	55 (14.6)
	6	24 (2.2)	24 (4.6)	- (-)	24 (6.4)
	7	18 (1.6)	18 (3.5)	- (-)	18 (4.8)
	8	13 (1.2)	13 (2.5)	- (-)	13 (3.4)
	9	4 (0.4)	4 (0.8)	- (-)	4 (1.1)
	10	2 (0.2)	2 (0.4)	- (-)	2 (0.5)
	Total	1114 (100)	517 (100)	140 (100)	377 (100)

d) Frequency (%) of PCGs answering sometimes or often for the 10 social needs, in order of highest to lowest frequency.

	All surveys		≥One unmet social needs	
In the past 12 months:	N	n (%)	N	n (%)
You worried that the food you bought would run out before you got the money or food stamps to buy more.	1112	342 (30.8)	515	342 (66.4)
The food you bought just didn't last and you didn't have money to get more.	1113	312 (28.0)	516	312 (60.5)
You were worried about or unable to pay your utility bills (gas, electric, phone, etc.).	1113	290 (26.1)	517	290 (56.1)
You were worried about or unable to find necessary household items (appliances, furniture, etc.).	1111	149 (13.4)	514	149 (29.0)
Are there any other things you have needed help with?	1097	137 (12.5)	504	137 (27.2)
You were unable to get child related resources (educational support for your children, childcare, diapers, clothing, car seats, cribs, etc.).	1111	130 (11.7)	514	130 (25.3)
Lack of transportation has kept you from meetings, work, running errands, or getting things needed for daily living.	1109	122 (11.0)	514	122 (23.7)
Lack of transportation has kept you from medical appointments or from getting medications.	1111	89 (8.0)	516	89 (17.2)
You felt physically or emotionally UNSAFE in your home.	1111	27 (2.4)	516	27 (5.2)
You were worried about where you and your family would spend the night.	1112	19 (1.7)	515	19 (3.7)

e) Demographics of PCGs and children: Categorical variables: For column headings, R% = row percent (percent of the row variable); C% = column % (percent of the column variable). Percents may not total exactly 100 due to rounding. P values are from chi-square tests (c) or Fisher's exact tests (f); na = not analyzed (insufficient memory for Fisher's exact test). P values <0.05 are bolded. For multiple comparisons between paired groups, P values were adjusted with Bonferroni corrections for 3 multiple comparisons. a: P<0.05 versus No unmet social needs; b: P<0.05 versus One unmet social need.

Variable	Level	All surveys n (C%)	No unmet social needs n (R%) (C%)	One unmet social needs n (R%) (C%)	≥Two unmet social needs n (R%) (C%)	P value
Relation to child	Mother	857 (79.9)	446 (52.0) (77.2)	107 (12.5) (81.1)	304 (35.5) (84.0)**a**	**0.001**
(4 levels)	Father	146 (13.6)	100 (68.5) (17.3)	18 (12.3) (13.6)	28 (19.2) (7.7)	f
	Grandparent	35 (3.3)	14 (40.0) (2.4)	4 (11.4) (3.0)	17 (48.6) (4.7)	
	Other	34 (3.2)	18 (52.9) (3.1)	3 (8.8) (2.3)	13 (38.2) (3.6)	
	Total	1072 (100)	578 (53.9) (100)	132 (12.3) (100)	362 (33.8) (100)	
PCG race	White	763 (72.0)	446 (58.5) (77.8)	91 (11.9) (71.1)**a**	226 (29.6) (63.0)**a**	**<0.001**
(3 levels)	Black	170 (16.0)	56 (32.9) (9.8)	25 (14.7) (19.5)	89 (52.4) (24.8)	c
	Other/multiracial	127 (12.0)	71 (55.9) (12.4)	12 (9.4) (9.4)	44 (34.6) (12.3)	
	Total	1060 (100)	573 (54.1) (100)	128 (12.1) (100)	359 (33.9) (100)	
PCG marital status (2 levels)	Married/unmarr couple	699 (65.3)	448 (64.1) (77.5)	79 (11.3) (60.8)**a**	172 (24.6) (47.5)**a,b**	**<0.001**
	Single/sep/divorce/wid	371 (34.7)	130 (35.0) (22.5)	51 (13.7) (39.2)	190 (51.2) (52.5)	c
	Total	1070 (100)	578 (54.0) (100)	130 (12.1) (100)	362 (33.8) (100)	
PCG education	≥ College grad	374 (34.9)	277 (74.1) (47.9)	48 (12.8) (36.6)	49 (13.1) (13.5)**a,b**	**<0.001**
(2 levels)	≤ AA/some college	697 (65.1)	301 (43.2) (52.1)	83 (11.9) (63.4)	313 (44.9) (86.5)	c
	Total	1071 (100)	578 (54.0) (100)	131 (12.2) (100)	362 (33.8) (100)	
Household income	≥$50,000	524 (50.7)	382 (72.9) (68.3)	61 (11.6) (48.0)**a**	81 (15.5) (23.3)**a,b**	**<0.001**
(2 levels)	<$50,000	510 (49.3)	177 (34.7) (31.7)	66 (12.9) (52.0)	267 (52.4) (76.7)	c
	Total	1034 (100)	559 (54.1) (100)	127 (12.3) (100)	348 (33.7) (100)	
Child's sex	Male	528 (47.6)	290 (54.9) (48.7)	65 (12.3) (46.8)	173 (32.8) (46.1)	0.715
	Female	581 (52.4)	305 (52.5) (51.3)	74 (12.7) (53.2)	202 (34.8) (53.9)	c
	Total	1109 (100)	595 (53.7) (100)	139 (12.5) (100)	375 (33.8) (100)	
Child race	White	654 (62.5)	392 (59.9) (68.8)	83 (12.7) (65.4)	179 (27.4) (51.1)**a**	**<0.001**
(3 levels)	Black	175 (16.7)	64 (36.6) (11.2)	25 (14.3) (19.7)	86 (49.1) (24.6)	c
	Other/multiracial	218 (20.8)	114 (52.3) (20.0)	19 (8.7) (15.0)	85 (39.0) (24.3)	
	Total	1047 (100)	570 (54.4) (100)	127 (12.1) (100)	350 (33.4) (100)	
Child's insurance	Public	614 (55.2)	237 (38.6) (39.8)	77 (12.5) (55.0)**a**	300 (48.9) (79.8)**a,b**	**<0.001**
	Private	483 (43.4)	353 (73.1) (59.2)	60 (12.4) (42.9)	70 (14.5) (18.6)	f
	None	15 (1.3)	6 (40.0) (1.0)	3 (20.0) (2.1)	6 (40.0) (1.6)	
	Total	1112 (100)	596 (53.6) (100)	140 (12.6) (100)	376 (33.8) (100)	

Child's insurance (2 levels, “none” excluded)	Public	614 (56.0)	237 (38.6) (40.2)	77 (12.5) (56.2)**a**	300 (48.9) (81.1)**a,b**	**<0.001** c
	Private	483 (44.0)	353 (73.1) (59.8)	60 (12.4) (43.8)	70 (14.5) (18.9)	
	Total	1097 (100)	590 (53.8) (100)	137 (12.5) (100)	370 (33.7) (100)	
Child's previous insurance	Public	559 (53.5)	210 (37.6) (37.6)	70 (12.5) (53.0)**a**	279 (49.9) (78.8)**a,b**	**<0.001** c
	Private	442 (42.3)	325 (73.5) (58.2)	55 (12.4) (41.7)	62 (14.0) (17.5)	
	None	43 (4.1)	23 (53.5) (4.1)	7 (16.3) (5.3)	13 (30.2) (3.7)	
	Total	1044 (100)	558 (53.4) (100)	132 (12.6) (100)	354 (33.9) (100)	
Child's overall health (4 levels)	Excellent	456 (41.6)	273 (59.9) (46.4)	69 (15.1) (50.4)	114 (25.0) (30.6)	na
	Very good	417 (38.0)	222 (53.2) (37.8)	42 (10.1) (30.7)	153 (36.7) (41.1)	
	Good	188 (17.1)	84 (44.7) (14.3)	16 (8.5) (11.7)	88 (46.8) (23.7)	
	Fair/poor	36 (3.3)	9 (25.0) (1.5)	10 (27.8) (7.3)	17 (47.2) (4.6)	
	Total	1097 (100)	588 (53.6) (100)	137 (12.5) (100)	372 (33.9) (100)	
Child's overall health (2 levels)	Excellent/very good/good	1061 (96.7)	579 (54.6) (98.5)	127 (12.0) (92.7)**a**	355 (33.5) (95.4)**a**	**<0.001** f
	Fair/poor	36 (3.3)	9 (25.0) (1.5)	10 (27.8) (7.3)	17 (47.2) (4.6)	
	Total	1097 (100)	588 (53.6) (100)	137 (12.5) (100)	372 (33.9) (100)	

f) Demographics of PCGs and Index Children: Continuous variables: IQR = interquartile range; SD = standard deviation. P values are from one-way analysis of variance (ANOVA) (oa), Welch ANOVA (wa), or Kruskal-Wallis one-way ANOVA on ranks (kw). Variables analyzed with kw are right skewed; variables analyzed with oa are approximately normally distributed. P values <0.05 are bolded. For multiple comparisons between paired groups, P values were adjusted with Bonferroni corrections for 3 multiple comparisons. **a**: P<0.05 versus No unmet social needs group; **b**: P<0.05 versus One unmet social need group.

Variable	Statistic	All surveys	No unmet social needs	One unmet social need	≥Two unmet social needs	P value
PCG age (years)	Mean (SD)	36 (10)	36 (10)	35 (8)	35 (10)**a**	**0.003**
	Median (IQR)	34 (11)	35 (12)	34 (11)	33 (12)	kw
	Range	18-75	18-75	21-58	20-73	
	n	1064	571	132	361	
Child's age (years)	Mean (SD)	6.4 (5.3)	6.4 (5.4)	6.3 (5.3)	6.6 (5.1)	0.672
	Median (IQR)	5.5 (9.1)	5.2 (9.2)	5.5 (9.1)	5.7 (9.3)	kw
	Range	0-18	0-18	0-18	0-18	
	n	1114	597	140	377	
