# Supplementary material for: Prevalence and Correlates of Families’ Unmet Social Needs in Pediatric Primary Care Settings
Source: Healthcare (Basel). 2026 Jun 12;14(12):1671. doi: 10.3390/healthcare14121671 (PMC13299676; doi:10.3390/healthcare14121671)
Supplement: Supplementary file 1 [file healthcare-14-01671-s001.zip › Waters_S2_Correlate Variables.pdf]

Supplementary Table S2: Summary of Survey correlates with unmet social needs. Categorical variables: For column headings, R% = row percent (percent of the row variable); C % = column % (percent of the column variable). Percents may not total exactly 100 due to rounding. P values are from chi-square tests (c) or Fisher's exact tests (f); na = not analyzed (insufficient memory for Fisher's exact test). P values <0.05 are bolded. For multiple comparisons between paired groups, P values were adjusted with Bonferroni corrections for 3 multiple comparisons. a: P<0.05 versus No unmet social needs group; b: P<0.05 versus One unmet social need group.

a) Maternal Social Support Index (MSSI), Social Capital (below vs. at/above median), depression screen, drug/alcohol, guns in home, bullets in home:

Variable	Level	All surveys n (C%)	No unmet social needs n (R%) (C%)	One unmet social needs n (R%) (C%)	≥Two unmet social needs n (R%) (C%)	P value
MSSI total score below vs at/above median of 23	At/above 23	579 (53.2)	393 (67.9) (67.3)	72 (12.4) (52.9)**a**	114 (19.7) (31.0)**a,b**	**<0.001**
	Below 23	509 (46.8)	191 (37.5) (32.7)	64 (12.6) (47.1)	254 (49.9) (69.0)	c
	Total	1088 (100)	584 (53.7) (100)	136 (12.5) (100)	368 (33.8) (100)	
Social Capital Scale total score below vs at/above median of 65	At/above 66	521 (52.0)	343 (65.8) (62.8)	61 (11.7) (51.7)	117 (22.5) (34.7)**a,b**	**<0.001**
	Below 66	480 (48.0)	203 (42.3) (37.2)	57 (11.9) (48.3)	220 (45.8) (65.3)	c
	Total	1001 (100)	546 (54.5) (100)	118 (11.8) (100)	337 (33.7) (100)	
Depression screen (neg/pos)	Negative	722 (67.2)	461 (63.9) (79.3)	97 (13.4) (74.0)	164 (22.7) (45.2)**a,b**	**<0.001**
	Positive	353 (32.8)	120 (34.0) (20.7)	34 (9.6) (26.0)	199 (56.4) (54.8)	c
	Total	1075 (100)	581 (54.0) (100)	131 (12.2) (100)	363 (33.8) (100)	
In the past week, you have felt depressed.	No days	660 (60.8)	422 (63.9) (72.5)	91 (13.8) (67.9)	147 (22.3) (39.8)**a,b**	**<0.001**
	Some days	370 (34.1)	152 (41.1) (26.1)	38 (10.3) (28.4)	180 (48.6) (48.8)	c
	Most days	55 (5.1)	8 (14.5) (1.4)	5 (9.1) (3.7)	42 (76.4) (11.4)	
	Total	1085 (100)	582 (53.6) (100)	134 (12.4) (100)	369 (34.0) (100)	
In past 12 m, had ≥2 weeks felt sad, blue, depressed, or lost pleasure doing things.	No	737 (67.9)	458 (62.1) (78.4)	98 (13.3) (73.7)	181 (24.6) (49.2)**a,b**	**<0.001**
	Yes	348 (32.1)	126 (36.2) (21.6)	35 (10.1) (26.3)	187 (53.7) (50.8)	c
	Total	1085 (100)	584 (53.8) (100)	133 (12.3) (100)	368 (33.9) (100)	
In lifetime, ≥2 years felt sad or depressed most days, even if you felt okay sometimes.	No	682 (62.8)	441 (64.7) (75.6)	80 (11.7) (60.6)**a**	161 (23.6) (43.4)**a,b**	**<0.001**
	Yes	404 (37.2)	142 (35.1) (24.4)	52 (12.9) (39.4)	210 (52.0) (56.6)	c
	Total	1086 (100)	583 (53.7) (100)	132 (12.2) (100)	371 (34.2) (100)	
Does anyone in your household have a drug or alcohol problem?	No	1076 (99.0)	578 (53.7) (99.3)	132 (12.3) (99.2)	366 (34.0) (98.4)	0.341
	Yes	11 (1.0)	4 (36.4) (0.7)	1 (9.1) (0.8)	6 (54.5) (1.6)	f
	Total	1087 (100)	582 (53.5) (100)	133 (12.2) (100)	372 (34.2) (100)	

b) Underinsurance variables:

Variable	Level	All surveys n (C%)	No unmet social needs n (R%) (C%)	One unmet social needs n (R%) (C%)	≥Two unmet social needs n (R%) (C%)	P value
Underinsured	No	999 (93.5)	565 (56.6) (97.9)	128 (12.8) (93.4)**a**	306 (30.6) (86.2)**a**	**<0.001**
	Yes	70 (6.5)	12 (17.1) (2.1)	9 (12.9) (6.6)	49 (70.0) (13.8)	c
	Total	1069 (100)	577 (54.0) (100)	137 (12.8) (100)	355 (33.2) (100)	
In past 12 m, delayed seeking medical care because of trouble paying	No	1054 (96.3)	582 (55.2) (98.8)	135 (12.8) (96.4)	337 (32.0) (92.3)**a**	**<0.001**
	Yes	40 (3.7)	7 (17.5) (1.2)	5 (12.5) (3.6)	28 (70.0) (7.7)	c
	Total	1094 (100)	589 (53.8) (100)	140 (12.8) (100)	365 (33.4) (100)	
In past 12 m, unable to make/keep appointment because of trouble paying	No	1072 (96.9)	588 (54.9) (99.0)	134 (12.5) (95.7)**a**	350 (32.6) (94.1)**a**	**<0.001**
	Yes	34 (3.1)	6 (17.6) (1.0)	6 (17.6) (4.3)	22 (64.7) (5.9)	f
	Total	1106 (100)	594 (53.7) (100)	140 (12.7) (100)	372 (33.6) (100)	
In past 12 m, unable to see specialist because of trouble paying	No	1074 (97.8)	587 (54.7) (99.3)	133 (12.4) (96.4)**a**	354 (33.0) (95.9)**a**	**<0.001**
	Yes	24 (2.2)	4 (16.7) (0.7)	5 (20.8) (3.6)	15 (62.5) (4.1)	f
	Total	1098 (100)	591 (53.8) (100)	138 (12.6) (100)	369 (33.6) (100)	
In past 12 m, unable to have test done because of trouble paying	No	1086 (98.7)	592 (54.5) (99.8)	138 (12.7) (99.3)	356 (32.8) (96.7)**a**	**<0.001**
	Yes	14 (1.3)	1 (7.1) (0.2)	1 (7.1) (0.7)	12 (85.7) (3.3)	f
	Total	1100 (100)	593 (53.9) (100)	139 (12.6) (100)	368 (33.5) (100)	
In past 12 m, unable to fill prescription because of trouble paying	No	1073 (97.4)	587 (54.7) (99.3)	139 (13.0) (99.3)	347 (32.3) (93.5)**a,b**	**<0.001**
	Yes	29 (2.6)	4 (13.8) (0.7)	1 (3.4) (0.7)	24 (82.8) (6.5)	f
	Total	1102 (100)	591 (53.6) (100)	140 (12.7) (100)	371 (33.7) (100)	
In past 12 m, unable to get other medical care because of trouble paying	No	1074 (97.9)	591 (55.0) (99.8)	135 (12.6) (97.8)	348 (32.4) (94.8)**a**	**<0.001**
	Yes	23 (2.1)	1 (4.3) (0.2)	3 (13.0) (2.2)	19 (82.6) (5.2)	f
	Total	1097 (100)	592 (54.0) (100)	138 (12.6) (100)	367 (33.5) (100)	
In past 12 m, child's health suffered because unable to pay for care (excludes “Don’t know”)	No	1078 (97.3)	591 (54.8) (99.5)	133 (12.3) (95.7)**a**	354 (32.8) (94.4)**a**	**<0.001**
	Yes	30 (2.7)	3 (10.0) (0.5)	6 (20.0) (4.3)	21 (70.0) (5.6)	f
	Total	1108 (100)	594 (53.6) (100)	139 (12.5) (100)	375 (33.8) (100)	
Getting care is easier, the same, or harder than 3 yrs ago (includes “Don’t know”)	Easier	100 (9.2)	56 (56.0) (9.6)	13 (13.0) (9.6)	31 (31.0) (8.4)**a**	**0.023**
	Stayed the same	759 (69.8)	424 (55.9) (72.9)	94 (12.4) (69.1)	241 (31.8) (65.3)	c
	Harder	129 (11.9)	51 (39.5) (8.8)	16 (12.4) (11.8)	62 (48.1) (16.8)	
	Don't know	99 (9.1)	51 (51.5) (8.8)	13 (13.1) (9.6)	35 (35.4) (9.5)	
	Total	1087 (100)	582 (53.5) (100)	136 (12.5) (100)	369 (33.9) (100)	
Getting care is easier, the same, or harder than 3 yrs ago (excludes “Don’t know”)	Easier	100 (10.1)	56 (56.0) (10.5)	13 (13.0) (10.6)	31 (31.0) (9.3)**a**	**0.006**
	Stayed the same	759 (76.8)	424 (55.9) (79.8)	94 (12.4) (76.4)	241 (31.8) (72.2)	c
	Harder	129 (13.1)	51 (39.5) (9.6)	16 (12.4) (13.0)	62 (48.1) (18.6)	
	Total	988 (100)	531 (53.7) (100)	123 (12.4) (100)	334 (33.8) (100)	

c) Children with Special Health Care Needs (CSHCN):

Variable	Level	All surveys n (C%)	No unmet social needs n (R%) (C%)	One unmet social needs n (R%) (C%)	≥Two unmet social needs n (R%) (C%)	P value
CSHCN group (8 levels)	Not CSHCN	723 (68.7)	426 (58.9) (74.7)	93 (12.9) (69.4)	204 (28.2) (58.6)	na
	Dependency only	52 (4.9)	28 (53.8) (4.9)	8 (15.4) (6.0)	16 (30.8) (4.6)	
	Service use only	61 (5.8)	31 (50.8) (5.4)	6 (9.8) (4.5)	24 (39.3) (6.9)	
	Funct limitations only	3 (0.3)	0 (0.0) (0.0)	0 (0.0) (0.0)	3 (100) (0.9)	
	Depend+Service	99 (9.4)	45 (45.5) (7.9)	16 (16.2) (11.9)	38 (38.4) (10.9)	
	Depend+Function	3 (0.3)	1 (33.3) (0.2)	0 (0.0) (0.0)	2 (66.7) (0.6)	
	Service+Function	54 (5.1)	19 (35.2) (3.3)	8 (14.8) (6.0)	27 (50.0) (7.8)	
	Depend+Serv+Funct	57 (5.4)	20 (35.1) (3.5)	3 (5.3) (2.2)	34 (59.6) (9.8)	
	Total	1052 (100)	570 (54.2) (100)	134 (12.7) (100)	348 (33.1) (100)	
CSHCN group (3 levels)	Not CSHCN	723 (68.7)	426 (58.9) (74.7)	93 (12.9) (69.4)	204 (28.2) (58.6)**a,b**	**<0.001**
	Depend and/or Service	212 (20.2)	104 (49.1) (18.2)	30 (14.2) (22.4)	78 (36.8) (22.4)	c
	Funct lim (w/wo Dep or Serv)	117 (11.1)	40 (34.2) (7.0)	11 (9.4) (8.2)	66 (56.4) (19.0)	
	Total	1052 (100)	570 (54.2) (100)	134 (12.7) (100)	348 (33.1) (100)	
CSHCN: none vs. ≥1 of Depend, Service, Funct limitations	Not CSHCN	723 (68.7)	426 (58.9) (74.7)	93 (12.9) (69.4)	204 (28.2) (58.6)**a**	**<0.001**
	CSHCN	329 (31.3)	144 (43.8) (25.3)	41 (12.5) (30.6)	144 (43.8) (41.4)	c
	Total	1052 (100)	570 (54.2) (100)	134 (12.7) (100)	348 (33.1) (100)	
CSHCN: dependency	No	868 (80.0)	491 (56.6) (83.5)	109 (12.6) (80.1)	268 (30.9) (74.2)**a**	**0.002**
	Yes	217 (20.0)	97 (44.7) (16.5)	27 (12.4) (19.9)	93 (42.9) (25.8)	c
	Total	1085 (100)	588 (54.2) (100)	136 (12.5) (100)	361 (33.3) (100)	
CSHCN: service use	No	800 (74.0)	463 (57.9) (79.4)	104 (13.0) (75.9)	233 (29.1) (64.5)**a,b**	**<0.001**
	Yes	281 (26.0)	120 (42.7) (20.6)	33 (11.7) (24.1)	128 (45.6) (35.5)	c
	Total	1081 (100)	583 (53.9) (100)	137 (12.7) (100)	361 (33.4) (100)	
CSHCN: functional limitations	No	983 (89.3)	548 (55.7) (93.2)	128 (13.0) (92.1)	307 (31.2) (82.1)**a,b**	**<0.001**
	Yes	118 (10.7)	40 (33.9) (6.8)	11 (9.3) (7.9)	67 (56.8) (17.9)	c
	Total	1101 (100)	588 (53.4) (100)	139 (12.6) (100)	374 (34.0) (100)	
