# Supplementary material for: Prevalence and Correlates of Families’ Unmet Social Needs in Pediatric Primary Care Settings
Source: Healthcare (Basel). 2026 Jun 12;14(12):1671. doi: 10.3390/healthcare14121671 (PMC13299676; doi:10.3390/healthcare14121671)
Supplement: Supplementary file 1 [file healthcare-14-01671-s001.zip › Waters_S3_MLR.pdf]

Supplementary Table S3: Multinomial Logistic Regression Results. Multinomial logistic regression predicting the odds of having one or ≥two unmet social needs versus no unmet social needs:The analysis used was multinomial logistic regression (no ordering of the dependent variable categories) rather than ordinal logistic regression because the required assumption of "parallel lines" for ordinal regression is violated by the data. Parallel lines means that the effect of an independent variable on the odds of falling in a category is equal across all categories of the dependent variable. This is also called the "proportional odds assumption."The sample size for the regression is 834 (74.9% of total sample size of 1114). 473 of 834 (56.7%) have 0 unmet social needs, 95 of 834 (11.4%) have one unmet social need, and 266 (31.9%) have ≥two unmet social needs. The multinomial logistic regression model correctly predicts 402/473 (85.0%) of those with 0 unmet social needs, 1/95 (1.1%) of those with 1 unmet social need, and 184/266 (69.2%) of those with ≥two unmet social needs (overall correct prediction = 70.4%). The AORs for each variable are adjusted for all other variables in the model (i.e. all other variables in the table below).

Variable	n	One unmet social need Adjusted odds ratio (95% confidence interval)	P value	≥Two unmet social needs Adjusted odds ratio (AOR) (95% confidence interval)	P value
# of people can count on					
1-person increase	834	0.99 (0.91, 1.08)	0.815	0.85 (0.78, 0.92)	**<0.001**
Underinsured					
Yes	45	2.23 (0.63, 7.93)	0.216	9.85 (4.05, 23.93)	**<0.001**
No	789	Reference	-	Reference	-
Household income					
< $50,000	390	1.98 (0.97, 4.03)	0.059	3.03 (1.72, 5.32)	**<0.001**
≥ $50,000	444	Reference	-	Reference	-
Depression screen					
Positive	273	1.10 (0.64, 1.88)	0.742	2.67 (1.80, 3.97)	**<0.001**
Negative	561	Reference	-	Reference	-
CSHCN					
CSHCN[1]	267	1.20 (0.68, 2.13)	0.526	1.89 (1.22, 2.92)	**0.004**
Not CSHCN	567	Reference	-	Reference	-
PCG race					
Black	111	1.87 (0.92, 3.77)	0.082	2.02 (1.15, 3.55)	**0.015**
Oher/multiracial	100	0.72 (0.34, 1.55)	0.402	0.69 (0.38, 1.24)	0.215
White	623	Reference	-	Reference	-
Relation to child					
Father	122	0.80 (0.41, 1.55)	0.505	0.51 (0.26, 0.96)	**0.038**
Grandparent	24	0.50 (0.08, 3.05)	0.453	0.78 (0.22, 2.81)	0.699
Other	23	0.59 (0.11, 3.09)	0.534	0.55 (0.17, 1.78)	0.315
Mother	665	Reference	-	Reference	-
PCG education					
≤ Associate/Some college	516	0.79 (0.44, 1.43)	0.439	1.72 (1.02, 2.90)	**0.043**
≥ College grad	318	Reference	-	Reference	-
Child's overall health					
Fair/poor	26	3.98 (1.19, 13.27)	**0.025**	3.10 (0.96, 10.01)	0.059
Good	136	0.46 (0.20, 1.09)	0.078	1.68 (0.95, 2.97)	0.073
Very good	319	0.72 (0.42, 1.21)	0.210	1.38 (0.88, 2.15)	0.158
Excellent	353	Reference	-	Reference	-

Social Capital Scale					
Below median of 66	389	1.54 (0.92, 2.58)	0.097	1.47 (0.97, 2.21)	0.068
At/above median of 66	445	Reference	-	Reference	-
PCG marital status					
Single/separ/divorced/widow	277	1.22 (0.67, 2.22)	0.518	1.38 (0.88, 2.16)	0.159
Married/unmarried couple	557	Reference	-	Reference	-
MSSI					
Below median of 23	379	1.32 (0.75, 2.32)	0.342	1.28 (0.81, 2.02)	0.290
At/above median of 23	455	Reference	-	Reference	-
Child's insurance					
Private	384	1.07 (0.51, 2.24)	0.853	0.86 (0.46, 1.59)	0.632
Public	450	Reference	-	Reference	-
PCG age (years)					
1-year increase	834	1.01 (0.98, 1.04)	0.603	1.00 (0.97, 1.02)	0.705

[1]CSHCN: positive for 1 or more of dependency, service use, or functional limitations.
